# Optimizing nutrient inputs by balancing spring wheat yield and environmental effects in the Hetao Irrigation District of China

**DOI:** 10.1038/s41598-022-26668-z

**Published:** 2022-12-29

**Authors:** Yuzhen Chen, Na Zhao, Yunfeng Hao, Xiaohong Li, Mingshou Fan, Xiaohua Shi, Liguo Jia

**Affiliations:** 1 College of Chemistry and Environment, Hohhot Minzu College, Hohhot, 010051 China; 2 College of Agronomy, Inner Mongolia Agricultural University, Hohhot, 010018 China; 3 Bayannur Academy of Agricultural and Animal Husbandry Sciences, Linhe, 015000 China

**Keywords:** Plant physiology, Agroecology

## Abstract

The Hetao Irrigation District is the primary spring wheat production region in China. However, overuse and unscientific use of chemical fertilizer have resulted in low nutrient use efficiency and potential risks to the environment. Balanced fertilization (BF), a 29.9–36.4% N fertilizer and 40% P fertilizer, was reduced, while a 72 kg K_2_O ha^−1^ K fertilizer was supplied and designed to resolve problems encountered during the field trial from 2019 to 2021. The results showed that the grain yield did not decrease significantly in the BF treatments compared in the local farmer practice (FP) treatment. The nitrogen fertilizer partial productivity (PFP_N_) and agronomic nitrogen efficiency (NAE_N_) increased 42.95–52.88% and 44.06–49.24% with BF compared to with the FP, respectively. Moreover, the BF treatments reduced nitrate leaching in the 0–100 cm soil layer and reduced the N surplus (N_sur_) to approximately 160 kg N per hectare per year, dramatically reducing the environmental risk. The yield maintenance and nitrogen use efficiency increases were attributed to the lower nitrogen concentrations in the seedlings and the higher apparent N translocation efficiency (TR) from the stems and sheaths after anthesis in the BF treatments than in the FP treatments. Considering the yield, nutrient use efficiency, and environmental and economic benefits comprehensively, the BF1 treatment was considered the optimal fertilization scheme for Hetao spring wheat production.

## Introduction

The Hetao Irrigation District is one of the three largest irrigation districts in China, where spring wheat accounts for over half of the total crop planting area^1,2^. The region has abundant light and heat resources, as well as water resources from the Yellow River, which are required for spring wheat growth and development. Local spring wheat is broadly appreciated throughout the country for its superior flour quality. However, flood irrigation has been the major irrigation mode for a long time, leading to not only wasted water resources but also leached fertilizer and runoff. The spring wheat is mainly cultivated as a single cropping locally. To obtain high yields and benefits, local farmers have increasingly used chemical fertilizers during spring wheat production. A recent investigation showed that the total fertilizer rates reached 1050 kg ha^−1^, especially nitrogen fertilizer, which reached 340 kg ha^−1^. It severely surpassed the 169 kg ha^−1^ recommended amount^3^. Overuse of chemical nitrogen fertilizer not only inhibits grain yield promotion but threatens environmental safety.

Overuse and unscientific management of nitrogen fertilizer has occurred widely in the crop system in China, which has led to soil acidification and cropland pollution during the last decade^4,5^. To address this issue, the Chinese government released the Action Plan for the Zero Increase of Fertilizer Use to ensure that the use of synthetic N fertilizer would no longer increase by 2020^6^. However, simply reducing the use of N fertilizer might decrease yield and is detrimental to food security^7^. Thus, different strategies need to be developed to reduce the nitrogen fertilizer rate in different regions and crop systems. Substituting mineral fertilizer with organic fertilizer is considered an important way to address the problem. A meta-analysis from Wei et al. (2020) showed that the optimal substitution rate for organic N was 40–60% in maize production, under which grain yield increased 4.2% and N leaching and runoff decreased by 26.9% at an equivalent N rate^8^. However, misuse and overuse of organic fertilizer might result in antibiotics or heavy metal pollution entering croplands and water bodies^9^.

A Nitrate Directive (ND) issued by the European Union was used to reduce nitrate leaching from agriculture through improved nutrient management. Balanced nitrogen application is an effective measure to reduce N losses^10^. A 33-year field experiment in North China showed that only applied nitrogen fertilizer resulted in a low wheat yield similar to the scenario with no fertilization, while combined N, P and K fertilization resulted in the highest grain yield comparable to manure and nitrogen fertilization^11^. Many studies have shown that balanced fertilization not only increases crop yield, but also promotes soil carbon retention and nutrient use efficiency^12–14^.

Fertilization imbalance is very serious in spring wheat production in the Hetao Irrigation District, and almost all smaller-holder farmers do not apply potash fertilizer^3^. A long-term deficiency in potassium supplementation might lead to an imbalance in soil mineral elements, affecting nitrogen absorption and resulting in low nutrient use efficiency. Therefore, balanced fertilization by replenishing potash fertilizer in spring wheat production should be a potential mechanism for decreasing nitrogen fertilizer usage and promoting its use efficiency. Under such conditions, developing a green and efficient fertilization method is an essential process for ensuring the green and sustainable development of local spring wheat production. In this project the balanced fertilization scheme involved, by decreasing the application of nitrogen fertilizer and phosphate fertilizer and supplementing with suitable potash fertilizer, to reconstruct a green and highly efficient planting mode for local spring wheat production.

## Materials and methods

### Research site description

**Figure 1:**
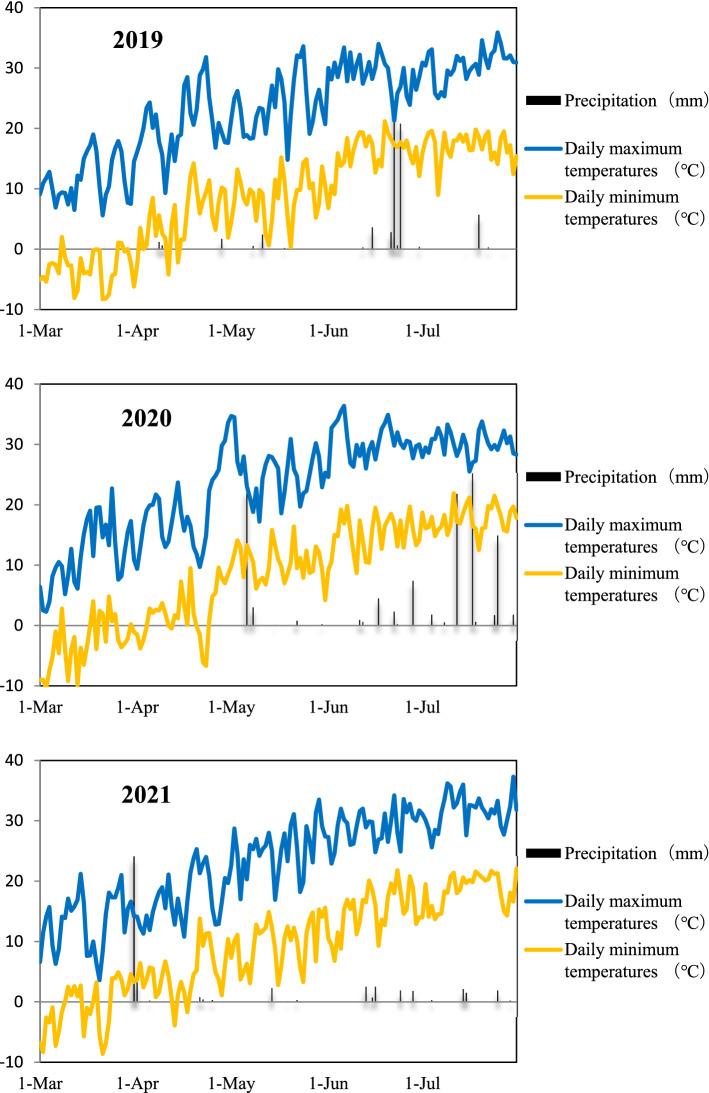
Daily maximum temperature, daily minimum temperature and precipitation during the growth period (March–July) of spring wheat from 2019 to 2021 in the field experiment at Linhe, Inner Mongolia, China.

A 3-year stationary field experiment was conducted at the Yuanziqu experimental station of the Bayannur Academy of Agricultural and Animal Husbandry Sciences (40° 90′ N, 107° 17′ E), Linhe, Inner Mongolia, China, from 2019 to 2021. The site has a continental monsoon climate typical of the northern mid-temperate zone, with a mean annual temperature from 3.7  to  7.6 °C, and the potential evaporation is 2200–2400 mm^15^. The total precipitation during the wheat growth period (March–July) was 66 mm, 110 mm and 47 mm in 2019, 2020, and 2021, respectively. Daily air temperature and precipitation during the field trial period are presented in Fig. 1.

The soil at the experimental site is a silt loam. The major physical and chemical properties of the 0–20 cm soil layer at the experimental site in 2019 were as follows: a bulk density of 1.48 g cm^−3^, pH 8.3, organic matter content of 15.49 g kg^−1^, total N concentration of 1.20 g kg^−1^, nitrate (NO_3_^−^ N) concentration of 3.98 mg N kg^−1^, Olsen-P concentration of 32.3 mg P kg^−1^ and available K concentration of 180.0 mg K kg^−1^.

### Experimental design and field management

A local popular spring wheat (*Triticum aestivum *L.) cultivar, Yongliang No. 4, was used in the trials. The sowing dates were 20 March, 16 March and 13 March in 2019, 2020 and 2021, respectively; the harvest dates were 15 July, 15 July and 5 July in 2019, 2020 and 2021, respectively. A total of 375 kg ha^−1^ wheat seeds were sown at a depth of 5 cm.

Five fertilization treatments were set in the three consecutive experimental years, including the control (CK), farmer practice (FP) and three balanced fertilization treatments (BF1, BF2 and BF3), as presented in Table 1. The P and K fertilizers as single superphosphate and K_2_SO_4_ were basal dressed, respectively. Both basal- and top-dressing of N fertilizer as urea were applied as shown in the research program. The basal applications occurred during sowing, and the remaining N fertilizer was top-dressed at the tillering stage (Table 1). The experimental plot was 10 m × 7 m with 13 cm row spacing and a buffer zone of 1 m between plots. The plots were laid out in a completely randomized block design with three replications.

**Table 1:** Fertilization regimes of the different treatments in the 2019–2021 field trial.

Treatment	Basal dressing (kg ha^−1^)			Top dressing (kg ha^−1^)
	N	P_2_O_5_	K_2_O	N
FP	102.0	172.5	0	172.5
CK	0	111.0	72.0	0
BF1	43.5	111.0	72.0	149.0
BF2	43.5	111.0	72.0	138.0
BF3	43.5	111.0	72.0	131.1

Every plot was ridged around its border to ensure the uniformity of irrigation. Flood irrigation from Yellow River water was performed according to the local policy and farmer practices. Irrigation water (30 mm) was applied at the tillering, jointing, heading and grain filling stages of spring wheat in 2019–2021. Disease, weed, and pest control, as well as other management, were performed according to local standard methods.

### Sampling and sample analysis

Three 50 cm-long rows of spring wheat plants were selected randomly and pulled from each plot, from which 10 large, middle and small seedlings were picked out during each sampling effort. Then, the roots were cut off from the junction between the root and the stem, two plant parts (leaves and stems) before the heading stage, three plant parts (leaves, stems and spikes) at the heading stage, and four plant parts (leaves, stems, glumes and grains) at the grain filling stages and maturity were separated and pooled. The samples were dried for 30 min at 105 °C and then at 80 °C in an oven (DHG-9070A, China) until they reached a constant weight; the dry weight was then measured.

The N concentrations in leaves, stems, spikes and grains of spring wheat were measured with three replications depending on the crop stage, following the Kjeldahl procedure using an element analyzer (Vario El cube, Elementar, Germany).

Three soil cores containing 0–100 cm of soil were taken from each plot using an auger at the harvest of spring wheat each year. The soil samples of each 20 cm layer were collected separately and sealed immediately in a marked plastic bag. The extracts were immediately measured for nitrate-N concentration as described by Dai et al*.* (2015) with a continuous flow analyzer (SKALAR SAN^++^, Netherlands)^16^. The soil nitrate-N concentration was expressed on the basis of the oven-dried soil.

Grain yield was evaluated at maturity by selecting two 2 m^2^ (avoiding border rows) randomly and harvested. A fresh weight of ∼ 1 kg of grain from each plot was weighed in the field, and the water content from each plot was oven dried for the calculation. The actual yield was adjusted by a grain water content of 13%^17^. Grains per spike, 1000-grain weight and spike number were determined at three 50-cm sites sampled randomly from each plot.

### Calculation methods

To clarify the effect of nitrate residue in the soil under balanced fertilization, the amount of soil nitrate-N (AN, kg N ha^−1^) in each layer was expressed as:

$${\text{AN}} = \left( {{\text{Ti}}\;*\;{\text{Di}}\;*\;{\text{Ci}}} \right)/10$$

where Ti is the soil layer thickness (cm), Di is the soil bulk density (g cm^−3^), Ci is the soil nitrate concentration (mg N kg^−1^) of the corresponding layer, and 10 is the conversion coefficient^16^. The AN of 0–20, 20–40, 40–60, 60–80 and 80–100 cm soil layers were recorded and measured, respectively.

Nitrogen accumulation in the vegetative organs and their distribution into the grain were investigated. Based on the dry weight and corresponding measured N concentration in the different organs, apparent N translocation (TA, kg ha^−1^) and apparent N translocation efficiency (TR, %) were calculated as proposed by Cox et al.^18^ as follows:

$$\begin{aligned} {\text{TA}} & {\text{ = H}}_{{\text{N}}} - {\text{M}}_{{\text{N}}} \\ {\text{TR}} & = {\text{TA/H}}_{{\text{N}}} *100 \\ \end{aligned}$$

where H_N_ is the N assimilation in leaves or stems prior to anthesis (kg ha^−1^), M_N_ is the N assimilation in leaves or stems at maturity (kg ha^−1^).

Two parameters of nitrogen use efficiency of spring wheat, nitrogen fertilizer partial productivity (PFP_N_, kg/kg) and agronomic nitrogen efficiency (NAE_N_, kg kg^−1^) were determined using the following formulas:

$$\begin{aligned} {\text{PFP}}_{{\text{N}}} & = {\text{ Y}}_{N\; \, fertilizer} /{\text{N}}_{rate} \\ {\text{NAE}}_{{\text{N}}} & = \, \left( {{\text{Y}}_{{N\;fertilizer \, {-}}} {\text{Y}}_{blank} } \right)/{\text{N}}_{rate} \\ \end{aligned}$$

where Y_*N fertilizer*_ is the grain yield of the plot with dressed N fertilizer (kg ha^−1^), Y_*blank*_ is the grain yield of the plot without dressed N fertilizer (kg ha^−1^), and N_*rate*_ is the N rate of the dressed fertilizer plot (kg ha^−1^). Three measurements for each treatment was recorded and calculated.

Two key indicators were chosen to evaluate the risk of N losses as described by Li et al. (2020)^7^, including N surplus (kg N per hectare per year, N_sur_) and N input (kg N per hectare per year, N_input_). The N surplus was used to evaluate the risk of N losses and the N input to guide farmers’ fertilization practices directly. The detailed calculation is as follows:

$$\begin{aligned} {\text{N}}_{{{\text{sur}}}} & = {\text{ N}}_{{{\text{fer}}}} + {\text{ N}}_{{{\text{dep}}}} + {\text{ N}}_{{{\text{fix}}}} - {\text{ N}}_{{{\text{har}}}} \\ {\text{N}}_{{{\text{input}}}} & = {\text{ N}}_{{{\text{har}}}} + {\text{ N}}_{{{\text{sur}}}} + {\text{ soil N change in stock }}\;\;\;( \approx 0{\text{ in long run}}) \\ \end{aligned}$$

where N_fer_, N_dep_ and N_fix_ represent N from fertilizer, atmospheric deposition and biological fixed N, respectively. Seed N was negligible as it was present in a very small amount compared to the fertilization input^19^. The total N deposition of spring wheat field and biological N fixation were adopted according to Li et al. (2020). N_har_ refers to the harvested N in spring wheat.

### Economic analysis

The inputs into local spring wheat production included chemical fertilizer, irrigated water, agricultural chemicals, seed, mechanical effort and labor costs, while income was obtained from the grain and wheat straw. The net income was determined from the difference between the total output and total input. The irrigated water, agricultural chemicals, seed, mechanical effort and labor costs were the same for the different treatments. The prices of the input and output materials were determined according to the average local market prices, and fluctuations were not considered among years.

### Statistical analyses

The results were analyzed using SPSS software (version 19.0; SPSS Inc., Chicago, IL, USA). Analysis of variance (ANOVA) and the least significant difference (LSD) test were used, and a *P* value of 0.05 was considered significant.

## Results

### Grain yield and dry matter accumulation

**Figure 2:**
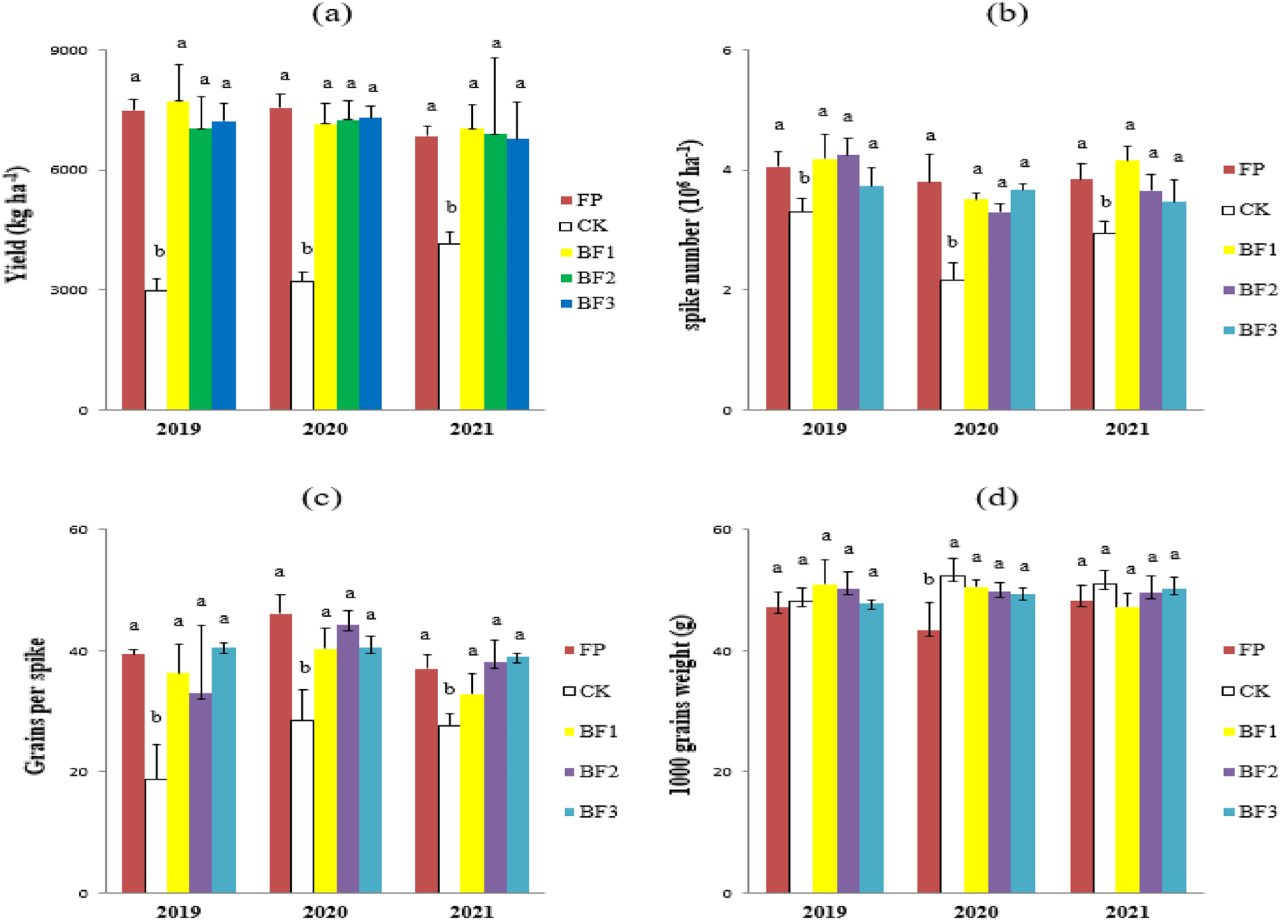
Effects of different balanced fertilization treatments on (**a**) yield, (**b**) spike number, (**c**) grains per spike and (**d**) 1000 grain weight of spring wheat from 2019 to 2021. The data are the means and standard errors of three independent measurements. Values within a separate year with the same letter are not significant at the *P* = 0.05 level. FP, CK and BF indicate farmer practice, control and balanced fertilization, respectively.

The balanced fertilization treatments (BFs) reduced N fertilization by 29.9–36.4% (BF1 to BF3) and P fertilization by 40%, while supplying 72 kg K_2_O ha^−1^ of K fertilizer. The on-farm field experiment showed that compared to the farmer practices (FP), the BFs did not decrease the grain yield of spring wheat. The grain yields of both the BF and FP treatments were significantly higher than those without N fertilizer (CK). There was no significant difference was exhibited among the three BF treatments (Fig. 2a). The spike number per hectare and grains per spike exhibited consistent tendencies with grain yield among the different treatments (Fig. 2b,c). There was no significant difference in the 1000-grain weight of spring wheat among all treatments except in 2020 (Fig. 2d).

**Figure 3:**
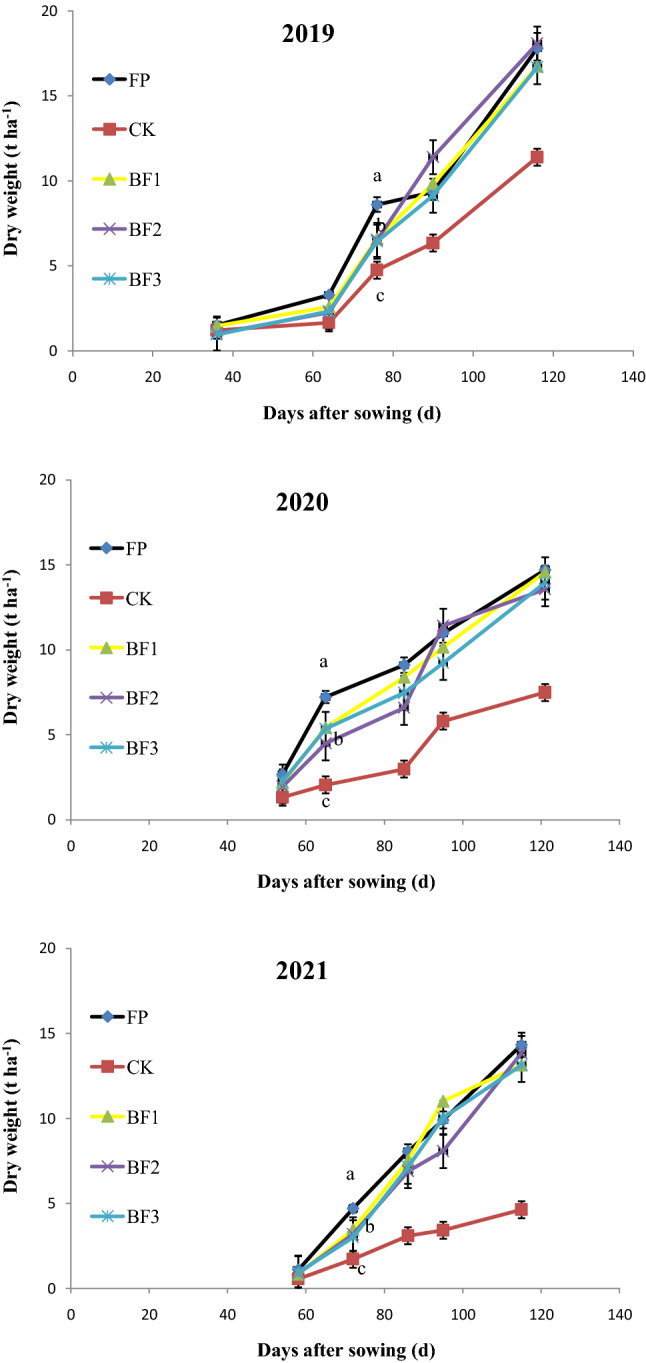
Dry matter accumulation of aboveground parts for different fertilization treatments in (**a**) 2019, (**b**) 2020 and (**c**) 2021. The data are the means and standard errors of three independent measurements. Vertical bars represent ± standard error of the mean (n = 3) where these exceed the size of the symbol.

Consistent with the changes in grain yield, the dry matter weight of the aboveground parts also exhibited higher levels in the BF and FP treatments than in the control during all developmental stages (Fig. 3). The dry matter accumulation was also higher during the early developmental period, before 80 days after sowing, in the FP treatment than in the BF treatment. This difference disappeared at later developmental stages until maturity. The pattern was similar among the consecutive 3-year field experiments (Fig. 3).

### Nitrogen use efficiency

Compared to in the farmer practices, in the BF treatments, 29.89–36.39% (BF1 to BF3) of nitrogen fertilizer was saved. The nitrogen use efficiency, PFP_N_ and NAE_N_, were dramatically higher in the BF treatments than in the FP treatment. With the reduction in the nitrogen rate in the BF treatments, the nitrogen use efficiency exhibited an increasing tendency. The PFP_N_ and NAE_N_ increased by an average of 42.95–52.88% and 44.06–49.24% in the BF treatments, compared to those in the farmer practice treatment in 2019–2021, respectively (Table 2).

**Table 2:** Nitrogen use efficiency of wheat among the different treatments.

Year	Treatments	Saved nitrogen fertilizer (%)	PFP_N_ (kg kg^−1^)	Increased PFP_N_ (%)	NAE_N_ (kg kg^−1^)	Increased NAE_N_ (%)
2019	FP	–	27.27 c	–	16.38 c	–
	BF1	29.89	40.12 a	47.11	24.59 a	50.09
	BF2	33.88	38.73 b	42.02	22.26 b	35.90
	BF3	36.39	41.29 a	51.40	24.17 a	47.54
2020	FP	–	27.50 c	–	15.78 c	–
	BF1	29.89	37.15 b	35.09	20.42 b	29.40
	BF2	33.88	39.94 ab	45.25	22.21 ab	40.73
	BF3	36.39	41.79 a	51.96	23.35 a	47.98
2021	FP	–	24.94 c	–	9.83 b	–
	BF1	29.89	36.57 b	46.64	15.01 a	52.67
	BF2	33.88	38.02 a	52.44	15.15 a	54.15
	BF3	36.39	38.73 a	55.29	14.96 a	52.20

FP and BF indicate farmer practice and balanced fertilization, respectively.

### Nitrogen absorption and translocation

To investigate the possible reasons that the BF treatments affected yield maintenance by dramatically reducing the nitrogen fertilizer rate, nitrogen absorption and translocation were determined. The concentration of nitrogen decreased gradually in the leaves and stems from the tillering stage to maturity and was also reduced in grains from the filling stage to maturity, as shown in the 2019 data. However, a more severe decrease in the nitrogen concentration was found in the BF treatments than in the FP treatment. In particular, the leaf nitrogen concentration in the BF treatments was almost half that in the FP treatment at maturity. In the stems and grains, the concentration of nitrogen at maturity was also significantly lower than that in the FP treatment (Table 3). A similar pattern was also found in 2020 and 2021 (data not shown).

**Table 3:** Nitrogen concentration (g kg^−1^ DW) in the leaves, stems and grains of the different treatments at different growth stages in 2019.

Organ	Treatment	Tillering	Jointing	Heading	Grain Filling	Maturity
Leaf	FP	18.84 a	19.73a	18.26 a	12.06 a	6.19 a
	CK	11.74 b	8.71 c	9.31 c	5.59 d	2.90 c
	BF1	19.35 a	16.16 b	14.95 b	11.12 b	3.99 b
	BF2	17.82 a	17.72 b	13.88 b	11.51 b	3.15 b
	BF3	17.49 a	15.55 b	13.55 b	9.22 c	3.43 b
Stem	FP	18.30 a	11.37 a	5.88 a	5.94 a	2.87 a
	CK	9.32 c	4.58 d	4.53 b	1.57 d	1.91 b
	BF1	16.09 b	11.15 a	5.40 a	4.19 b	1.54 c
	BF2	15.66 b	9.41 b	5.76 a	3.06 c	2.11b
	BF3	15.40 b	7.19 c	4.82 b	2.88 c	1.76 bc
Grain	FP				10.27 a	10.01 a
	CK				7.26 d	6.86 d
	BF1				9.17 b	8.98 b
	BF2				9.34 b	7.85 c
	BF3				8.45 c	7.02 cd

FP, CK and BF indicate farmer practice, control and balanced fertilization, respectively. Values within a column with different letters are significant at the *P* = 0.05 level.

**Figure 4:**
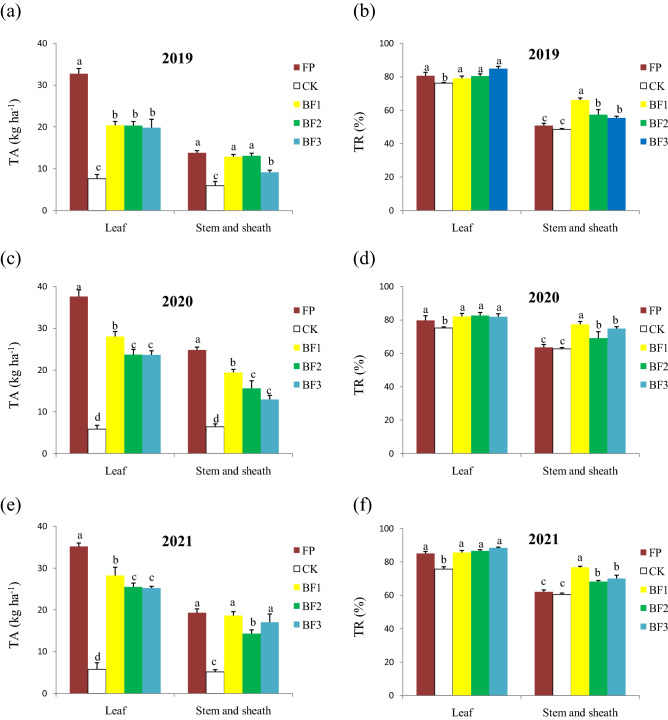
Effects of different balanced fertilization treatments on (**a**,**c**,**e**) apparent N translocation and (**b**,**d**,**f**) its efficiency in spring wheat from 2019 to 2021. The data are the means and standard errors of three independent measurements. Values within a cluster column with different letters are significant at the *P* = 0.05 level. TA and TR indicate apparent N translocation and apparent N translocation efficiency, respectively.

The apparent N translocation (TA) in the leaves was the highest for the FP treatment, and then for the BF treatments, and the lowest TA was in the CK; BF1 exhibited a higher TA level than BF2 and BF3 except for that in 2019. The TA from the stem and sheath showed a similar pattern to that in the leaf among the different treatments. Only some BF treatments exhibited the same level as that in the FP treatment during the 3-year field experiments (Fig. 4a,c,e). There was no significant difference in apparent N translocation efficiency (TR) in the leaves among the FP and BF treatments, although it was still the lowest in the CK. However, a significantly higher TR of the stem and sheath occurred in the BF treatments than in the FP treatment; the TR of the FP treatment was equal to that of the CK treatment (Fig. 4b,d,f).

### Evaluation of N losses

**Figure 5:**
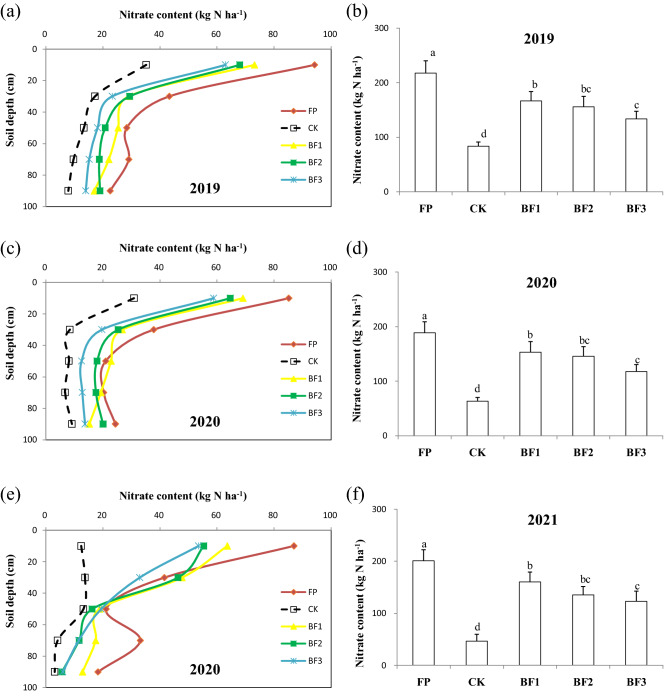
Changes in (**a**,**c**,**e**) different layers and (**b**,**d**,**f**) total NO_3_^−^ N content of 0–100 cm soil at spring wheat harvest for different fertilization treatments from 2019 to 2021. The data are the means and standard errors of three independent measurements. Values with different letters are significant at the *P* = 0.05 level.

Nitrogen leaching as NO_3_^−^ has been proposed as an important means of N loss during spring wheat production due to local flood irrigation habits. The average NO_3_^−^ residue at 0–100 cm at harvest was 202.5 kg ha^−1^ in the FP treatment. It was 160.2, 145.7 and 124.9 kg ha^−1^ in the BF1, BF2 and BF3 treatments, respectively, and it was only 64.5 kg ha^−1^ in the CK. The main difference in the NO_3_^−^ residue was in the 0–20 and 20–40 cm soil layers in 2019 and 2020 and in the 0–20 and 60–80 cm soil layers in 2021 (Fig. 5a,c,e). There was a significant difference in the NO_3_^−^ residue among the treatments, with the highest occurring in the FP treatment and the lowest in the CK, and that in BF1 was higher than that in the BF3 treatment (Fig. 5b,d,f).

Nitrogen input (N_input_), which considered fertilizer, atmospheric deposition and biological fixation as well as N surplus (N_sur_), was used to evaluate the nitrogen status in the spring wheat production system. A positive correlation between field N_input_ and N_sur_ was exhibited in the three experimental years, and both were highest in the FP treatment but lowest in the BF3 treatment. The N_sur_ reached 240.52 kg N per hectare per year in the FP treatment, which was dramatically reduced to approximately 160 kg N per hectare per year in the BF treatments (Table 4).

**Table 4:** Nitrogen input and surpluses of spring wheat among the different treatments.

Treatments	N_input_ (kg ha^−1^ year^−1^)	N_har_ (kg ha^−1^ year^−1^)				N_sur_ (kg ha^−1^ year^−1^)			
		2019	2020	2021	Ave	2019	2020	2021	Ave
FP	314.50	74.96	77.55	69.44	73.98	239.54	236.95	245.06	240.52
BF1	232.45	69.37	64.86	63.55	65.92	163.08	167.59	168.90	166.53
BF2	221.50	55.23	63.21	57.19	58.54	166.27	158.29	164.31	162.96
BF3	214.60	50.63	57.19	50.25	52.69	163.97	157.41	164.35	161.91

FP and BF indicate farmer practice and balanced fertilization, respectively.

### Revenue analysis

The irrigated water, agricultural chemicals, seed, mechanical effort and labor costs were the same for the different treatments at 1200 RMB ha^−1^, 300 RMB ha^−1^, 1875 RMB ha^−1^, 4200 RMB ha^−1^ and 3000 RMB ha^−1^, respectively. The cost of the chemical fertilizer was 1942.5 RMB ha^−1^, 1071 RMB ha^−1^, 1830 RMB ha^−1^, 1784.4 RMB ha^−1^ and 1755.9 RMB ha^−1^ in the FP, CK, BF1, BF2 and BF3 treatments, respectively. The price of harvested grain and straw was 2.8 RMB kg^−1^ and 0.53 RMB kg^−1^, respectively. The average net income of FP, CK, BF1, BF2 and BF3 at 3 years was 10,791.40 RMB ha^−1^, 366.54 RMB ha^−1^, 10,820.57 RMB ha^−1^,10,128.78 RMB ha^−1^ and 9763.86 RMB ha^−1^, respectively (Table 5). The net income of the BF1 treatment was higher than that of the farmer practices treatment and was the highest among all treatments. The lowest net income was found in the control without nitrogen fertilizer application, and it was especially negative in 2019.

**Table 5:** Net income (RMB ha^−1^) of spring wheat production among different treatments.

Year	FP	CK	BF1	BF2	BF3
2019	9558.85	− 2884.25	10,605.53	9327.97	8763.55
2020	12,002.62	431.57	10,592.39	10,499.91	10,353.60
2021	10,812.71	3552.29	11,263.81	10,558.47	10,174.42
Average	10,791.40	366.54	10,820.57	10,128.78	9763.86

FP, CK and BF indicate farmer practice, control and balanced fertilization, respectively.

## Discussion

China is facing the dual challenges of ensuring food security and environmental sustainability. The Hetao Irrigation District is one of the main and high-quality production regions of spring wheat in China. However, misunderstanding the fertilizer effects of local farmers in spring wheat production has resulted in the overuse of synthetic fertilization becoming increasingly serious. In particular, the input of chemical nitrogen fertilizer has severely exceeded the spring wheat requirement and recommended amount^3,20^. At the same time, potassium as an important macroelement has long been neglected, and most farmers don't use potash fertilizer in spring wheat production. Overused nitrogen is not absorbed by plants but released into the environment, resulting in an immense risk to local ecological safety.

Balanced fertilization by reducing nitrogen and supplementing with potash fertilizer is a potential effective way to maintain yield and reduce environmental risk in local spring wheat production. Based on the guidance of the 'Action Plan for the Zero Increase of Fertilizer Use' and the nutrient requirements of spring wheat^6,21^, dramatically reduced N and P fertilizer and supplementation with K fertilizer were used to investigate the yield and environmental effects in this study. The results of the 3-year stationary field experiment showed that no nitrogen fertilizer dressing resulted in a very low yield, while balanced fertilization with reduced N fertilizer (29.9–36.4%) and 40% P fertilizer did not significantly decrease grain yield (Fig. 2a). Therefore, it is not difficult to understand that in comparison to other fertilization regimes, balanced fertilization exhibits higher nitrogen use efficiency (expressed as PFP_N_ and NAE_N_, Table 2).

The reduction in nitrogen and phosphate fertilizer resulted in relatively lower dry matter accumulation before the jointing stage in the BP treatments, although when it was supplemented with potash fertilizer, dry matter increased in the FP treatment from the heading stage (Fig. 3). Previous studies also showed that postanthesis productivity improvement is more important for wheat yield formation^22–24^. Especially under high yield conditions, postanthesis photosynthetic capacity and DM mobilization play an essential role in final grain filling. The spring wheat yield level was not low in the farmer practice treatment at the Hetao Irrigation District. Therefore, rapid dry matter accumulation after anthesis contributes to grain yield maintenance under balanced fertilization modes.

Although the dry matter did not decrease at postanthesis, nitrogen absorption was reduced in the seedlings in the BF treatments (Table 3). Compared to the dramatic reduction in dry matter in the CK, in the BF treatments, the concentration of nitrogen in the leaves and stems was sufficient for normal growth and metabolism. In fact, the seedlings did not exhibit any nitrogen-deficiency phenotypes in the field in the BF treatments. Another reason for yield maintenance might be attributed to the higher nitrogen translocation efficiency (TR) from the stem and sheath to grain after anthesis (Fig. 4). Matter translocation promotion from the stem and sheath to the grain under stress conditions is an important mechanism for yield formation^25^. Although enough nitrogen was provided based on the wheat requirement in the BF treatments, the lower nitrogen concentration in the seedlings demonstrated there was no complete N absorption by the seedlings in the BF treatments. Theoretically, the nitrogen provided in the BF treatments was enough for normal seedling growth and development if there are no losses. The possible temporary deficiency of nitrogen could not be excluded due to losses induced by flood irrigation.

In fact, nitrate residue was found in the harvested soil in the local spring wheat production system. However, the nitrate content at 0–100 cm significantly decreased in the BP treatments in comparison to in the FP treatment (Fig. 5). Previous studies showed an average of 60–95 kg N ha^−1^ residual nitrate in the 0–100 cm soil layer under the recommended N fertilizer application rate^16,26^. Li et al. (2020) suggested that the maximum nitrate residue in the 0–90 cm soil layer is 90 kg N ha^−1^ in single cropping systems^7^. Our results showed that although balanced fertilization practices were implemented, NO_3_^−^ reached 125–160 kg N ha^−1^, which is still severely higher than the threshold level. Another nitrogen management-related environmental index, nitrogen surplus (N_sur_), also dramatically surpassed the benchmark even in the BF treatments, although it was much lower than that in the FP treatment (Table 4)^7,16^. Therefore, more elaborate nitrogen management strategies, such as fertilization frequency and fertigation techniques, need to be explored to increase nitrogen use efficiency in spring wheat production in the Hetao Irrigation District. Therefore, potential nitrogen savings still exist and need to be explored in the future.

The recommended fertilization scheme needs to consider not only yield and environmental risk but also local farmers’ economic benefits. Although there were no significant differences in yield among the three balanced fertilization treatments, the values were different. The net income of BF1 was higher than that of the other balanced fertilization schemes and farmer practices (Table 5). Moreover, BF1 exhibited less nitrate residue in the soil, although it was slightly higher than that in the other BF treatments. Thus, the BF1 treatment is recommended as the optimal fertilization scheme for Hetao spring wheat production under the present cultivation conditions. With improvements in the other agronomic practices, it is proposed that further balanced fertilizer and savings be applied.

## Conclusion

It could maintain spring wheat yield by reducing 29.9–36.4% N fertilizer and 40% P fertilizer via balanced fertilization in Hetao Irrigation District of China. Balanced fertilization with a 29.9% N fertilizer and 40% P fertilizer reduction, while a 72 kg K_2_O ha^−1^ K fertilizer supplied, increase nitrogen use efficiency and net income, but decrease nitrate leaching in soil. The beneficial effects of balanced fertilization are attributed to the lower nitrogen concentrations in the seedlings, and the higher apparent N translocation efficiency (TR) from the stems and sheaths after anthesis.

## Data Availability

The datasets used and/or analyzed during the current study are available from the corresponding author on reasonable request.
